# eHealth interventions for the prevention of depression and anxiety in the general population: a systematic review and meta-analysis

**DOI:** 10.1186/s12888-017-1473-1

**Published:** 2017-08-29

**Authors:** M. Deady, I. Choi, R. A. Calvo, N. Glozier, H. Christensen, S. B. Harvey

**Affiliations:** 1 School of Psychiatry, University of New South Wales, Sydney, NSW 2052 Australia; 2 Brain and Mind Centre, University of Sydney, Camperdown, NSW 2050 Australia; 3 School of Electrical and Information Engineering, University of Sydney, Sydney, NSW 2006 Australia; 4 Black Dog Institute, Randwick, NSW 2031 Australia; 5 St George Hospital, Kogarah, NSW 2217 Australia

**Keywords:** Depression, Anxiety, Prevention, Subclinical, eHealth, Mental disorder, Resilience

## Abstract

**Background:**

Anxiety and depression are associated with a range of adverse outcomes and represent a large global burden to individuals and health care systems. Prevention programs are an important way to avert a proportion of the burden associated with such conditions both at a clinical and subclinical level. eHealth interventions provide an opportunity to offer accessible, acceptable, easily disseminated globally low-cost interventions on a wide scale. However, the efficacy of these programs remains unclear. The aim of this study is to review and evaluate the effects of eHealth prevention interventions for anxiety and depression.

**Method:**

A systematic search was conducted on four relevant databases to identify randomized controlled trials of eHealth interventions aimed at the prevention of anxiety and depression in the general population published between 2000 and January 2016. The quality of studies was assessed and a meta-analysis was performed using pooled effect size estimates obtained from a random effects model.

**Results:**

Ten trials were included in the systematic review and meta-analysis. All studies were of sufficient quality and utilized cognitive behavioural techniques. At post-treatment, the overall mean difference between the intervention and control groups was 0.25 (95% confidence internal: 0.09, 0.41; *p* = 0.003) for depression outcome studies and 0.31 (95% CI: 0.10, 0.52; *p* = 0.004) for anxiety outcome studies, indicating a small but positive effect of the eHealth interventions. The effect sizes for universal and indicated/selective interventions were similar (0.29 and 0.25 respectively). However, there was inadequate evidence to suggest that such interventions have an effect on long-term disorder incidence rates.

**Conclusions:**

Evidence suggests that eHealth prevention interventions for anxiety and depression are associated with small but positive effects on symptom reduction. However, there is inadequate evidence on the medium to long-term effect of such interventions, and importantly, on the reduction of incidence of disorders. Further work to explore the impact of eHealth psychological interventions on long-term incidence rates.

## Background

Anxiety and depression, often termed ‘common mental disorders’ (CMD) because of their high prevalence rates in the general population [1], are associated with a substantial loss of quality of life for patients and their relatives [2, 3], increased mortality rates [4], high levels of service use, and enormous economic costs [5–8]. Major depression is currently the fourth leading cause of disease burden worldwide, and is expected to rank first in disease burden in high-income countries by the year 2030 [9].

To date, most of the effort to reduce the burden of these disorders has been targeted at ensuring treatment is given to those with existing disorders. Although effective treatments are available, cost-effectiveness models suggest that even in the unlikely event of optimal treatment being delivered to all cases, only 35 to 50% of the overall burden of common mental disorders would be alleviated [10]. As a result, policy makers and researchers have begun to consider strategies aimed at prevention [11, 12].

To date, however, there has been little consensus about what preventive strategies would be both effective and feasible to roll out to whole populations [13, 14]. Prevention programs can be universal (directed at an entire population), selective (only those at high risk), or indicated (only those with emerging symptoms) [15]. The relative effectiveness of the different types of prevention as they relate to mental health remains unclear. Additionally, levels of CMD symptomatology are often treated as proxy for the disorder itself (where full diagnostic interviews are not conducted) and, as such, prevention efforts in many cases target symptom reduction as a primary outcome with the view that reduction in symptomatology is likely to both reduce incidence and have positive effects on overall wellbeing and quality of life [16]. Although, it is now well accepted that it should be possible to prevent at least some new cases of CMD [17–19], it is important to consider the full spectrum of prevention models in evaluation. However, the cost associated with delivering most face-to-face psychological prevention programs has made large scale roll-outs unfeasible. The advent of technological innovation and eHealth interventions provides an opportunity to offer accessible, acceptable, easily disseminated low-cost interventions on a wide scale. It is known that certain eHealth interventions can have positive effects on symptoms of depression [20–23] and anxiety disorders [24, 25] among those with clinically relevant symptom levels. However, much less attention has been given to preventative interventions in this area. Similarly, in most eHealth trials no ceiling cut-offs are applied to inclusion criteria, making it difficult to determine how such interventions perform as prevention. Unwell populations are likely to differ in terms of motivation or responsiveness to intervention, compared to at-risk, subclinical, and general populations. As such, there is a need to look specifically at these non-clinical populations to precisely understand effective prevention interventions.

The aim of this systematic review and meta-analysis is to examine the effects of eHealth psychological interventions aimed at participants without clinically diagnosable common mental disorders on reduction of anxiety and depression symptoms and incidence. We also examined the relative effects of universal, selective/indicated prevention programs.

## Methods

### Search strategy

**Fig. 1:**
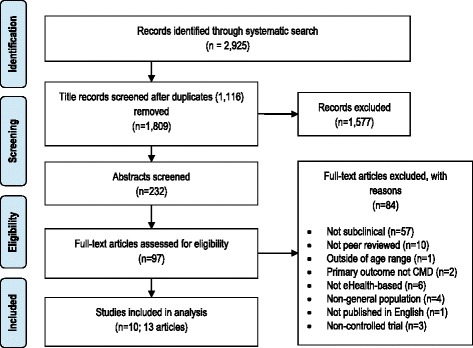
Search strategy

Consistent with methods detailed in the *Cochrane Collaboration’s Handbook* [26] and *PRISMA statement for systematic reviews* [27], the search strategy comprised two steps. First, a comprehensive literature search was conducted using the electronic databases PubMed, PsycINFO, EMBASE, and Cochrane library for relevant articles published from 2000 to January 2016. The search strategy was limited to these years due to the first internet trials focused on mental health appearing in the literature at this time [28]. A combination of keywords relating to mental health, prevention, eHealth, and randomized controlled trials were used (Table 1). Secondly, the reference lists of all included studies from the above strategy were also examined to identify any relevant publications that had not been considered and a final search of PubMed for related articles of all included studies. Finally, a Google Scholar search was conducted to search for any other relevant literature. Figure 1 summarizes the systematic search strategy.

**Table 1:** Search strategy terms

Mental health AND	Prevention AND	Study design AND	eHealth AND	Title search
depress^a^.tw.	prevent^a^.tw.	RCT.tw.	eHealth.tw.	prevent^a^.ti.
anxi^a^.tw.	resilienc^a^.tw.	efficacy.tw.	internet^a^.tw.	resilienc^a^.ti.
mood disorder.tw.	at-risk^a^.tw.	random allocation.tw.	online.tw.	at-risk^a^.ti.
common mental^a^.tw.	at risk^a^.tw.	effectiveness.tw.	app.tw.	at risk^a^.ti.
obsessive compulsive.tw.	early intervention^a^.tw.	exp randomized controlled trial/	self-directed/ self directed.tw.	early intervention^a^.ti.
panic.tw.	subsyndromal^a^.tw.	randomi^a^.tw.	web-based/web based.tw.	depress^a^.ti.
post-traumatic stress.tw.	subthreshold^a^.tw.	trial.tw.	smart-phone^a^/ smartphone^a^.tw.	common mental^a^.ti.
	subclinical^a^.tw.	controlled clinical trial/	mobile phone^a^.tw.	anxi^a^.ti.
		clinical trial/	cell phone^a^.tw.	subsyndromal^a^.ti.
			technology-assisted.tw.	subthreshold^a^.ti.
			mHealth	subclinical^a^.ti.
			mobile health.tw.	
			unsupported.tw.	
			unguided.tw.	
			self-help/ self help.tw.	
			self-guided/ self guided.tw.	
			app-based.tw.	

^a^Retrieves all possible suffix variations of the root word indicated

### Inclusion and exclusion criteria

The criteria used for inclusion in this review were: (a) Subclinical or nonclinical sample (or studies that split into subclinical/diagnosed), this required either a diagnostic tool at baseline or the use of a subclinical cut-off on a validated measure in order to exclude cases; (b) Population aged between 18 and 64 years; (c) Primary outcome either incidence or symptom reduction of common mental disorder (depressive or anxiety disorder); (d) eHealth-based psychological intervention; and (e) randomised controlled trial comparing the intervention to a control group.

Articles excluded from the review were (a) not peer-reviewed; (b) uncontrolled; (c) not published in English; (d) used a child/adolescent or elderly population; (e) used a non-general population (e.g., post-natal, comorbid, chronic pain—as these interventions are likely to be limited in generalisability to wider population. Tertiary/workplace populations were acceptable). When multiple publications from the same study population were available, we report data from the most recent/relevant publication.

### Data selection

Two researchers (M.D. and I.C.) independently analysed each title and abstract in order to ascertain their relevance. Agreement was substantial at 83% (Kappa = 0.62, SE of Kappa = 0.05; 95% CI: 0.52 to 0.73; *p* ≤ 0.001). The full texts of the remaining studies (including discrepancies) were similarly analysed by two researchers to exclude papers that did not meet inclusion criteria. Agreement was 93% (Kappa = 0.80, SE of Kappa = 0.09; 95% CI: 0.62 to 0.97; *p* ≤ 0.001). In order to achieve consensus, any disagreements were discussed and resolved.

### Data extraction

The criteria used for data extraction from studies were adapted from the Cochrane Collaboration’s Handbook: Systematic Reviews of Health Promotion and Public Health Interventions [26]. These criteria relate to the intervention sample characteristics, type of intervention (length, design, follow-up period, age-appropriateness), and outcome indicators. All data required for effect size calculation was entered into STATA version 12.0 [29]. Where additional information was required study authors were contacted using correspondence addresses on the study reports. All authors responded.

### Quality assessment

The quality of the identified studies was assessed using the Downs and Black checklist [30]. This scale was identified as appropriate for the present review as it was specifically developed for the domain of public health. The Downs and Black checklist has been associated with good criterion validity (*r* = 0.90) [31], good inter-rater reliability (*r* = 0.75) and has previously been used in similar reviews [32, 33]. The 27-item checklist is comprised of five subscales measuring reporting, external validity, internal validity, and power.

Minor modifications were made to the tool for use in this review. The scoring for question 27 on power was simplified to either zero or one, based on whether or not there was sufficient power in the study to detect a clinically significant effect (i.e., studies reporting power of less than 0.80 with alpha at 0.05 obtained a zero score). The maximum score for the modified checklist was 28 with all individual items rated as either yes (= 1) or no/unable to determine (= 0), with the exception of item 5, “Are the distributions of principals confounders in each group of subjects to be compared clearly described?” in which responses were rated as yes (= 2), partially (= 1) and no (= 0). Scores were grouped into four categories based on ranges: Excellent (26 to 28), good (20 to 25), fair (15 to 19) and poor (14 and less). These changes were in line with previous studies [33, 34]. Quality assessments were completed by two independent reviewers with 95% agreement (Kappa = 0.89, SE of Kappa = 0.03; 95% CI: 0.83 to 0.95; *p* ≤ 0.001). In order to achieve consensus, any disagreements were discussed and resolved.

### Statistical analysis

As reduction in symptoms was the primary outcome in all the eligible studies, the main analysis was conducted using symptoms of depression or anxiety as the outcome respectively. Secondary sub-analyses were conducted to compare different forms of prevention (universal vs selective/indicated). Both post-intervention and follow-up effects are reported separately. As all the studies measured depression/anxiety using varying psychometric scales, the effect size measure was represented by the standardized mean differences (SMD), which compares the scores of the treatment and control group post-intervention, with 95% CIs. The effect size was calculated by subtracting the average score of the intervention group from that of the control group, and dividing the result by the pooled standard deviations. A positive effect size indicates superior effects of intervention group compared to the control group. In a clinical treatment setting, effect sizes of 0.8, 0.5 and 0.2 are considered to be large, moderate and small, respectively [35]. At a population level, when considering prevention interventions, small effect sizes are considered relevant.

In the studies that included two intervention groups, SMD were computed for each treatment-control comparison, and the number of subjects in the control group was evenly divided among the intervention groups to ensure that each participant was only included once in the analysis. If more than one outcome measure was used (e.g., Beck Depression Index and Beck Anxiety Index) these studies were included in both analyses.

The meta-analyses were performed in Stata/IC release 12.1 [29] statistical programming software. For the outcome scores, the pooled mean effect sizes are expressed as SMD with 95% confidence intervals (95% CI). To compare the dichotomous outcome variables (i.e. incidence rates), the pooled effects were presented as risk ratios (RR) with 95% CIs. The studies were weighted by the inverse-variance method. As considerable heterogeneity due to population and methodological diversity was expected, we calculated pooled effect size estimates using the random effects model (REM). The REM is a more conservative approach that assumes that all studies are estimating different effects resulting from variations in factors such as study population, sampling variation within and between studies, and as a result produces wider confidence intervals [36, 37].

To test for heterogeneity, effect sizes were measured using Cochran’s Q-statistic, for which a *P* < 0.1 was regarded as significant heterogeneity [36]. As the Cochran’s test only indicates the presence of heterogeneity and not its magnitude, we also reported the I^2^ statistic, which estimates the percentage of outcome variability that can be attributed to heterogeneity across studies. An I^2^ value of 0% denotes no observed heterogeneity, whereas, 25% is “low”, 50% is “moderate” and 75% is “high” heterogeneity [38].

Publication bias occurs when the published studies are unrepresentative of all conducted studies due to the tendency to submit or accept manuscripts on the basis of the strength or direction of the results [39]. We examined this form of bias through a funnel plot with the SMD plotted against the SMD standard error. Due to the limited number of studies included, the presence of asymmetry can be difficult to determine by inspection of the funnel plot, thus, Egger’s linear regression model was used to statistically test for asymmetry [40].

## Results

### Overview of search results and included studies

The detailed search in all databases identified a total of 1808 titles (following the removal of duplicates). One additional article was identified; this paper was a recent follow-up study to an included paper [41], resulting in a total of 1809 articles. After the independent selection process, 13 articles were identified as relevant to the research question and included in the analysis. Of these 13, three were follow-up papers to included studies, meaning there were 10 independent samples with a total of 4522 (relevant) participants. Summaries of the 10 independent samples are provided in Table 2.

**Table 2:** Study characteristics

Study	Sample/eligibility	Conditions	Support	Intervention	Follow-up period (rate)	Outcome	Conclusions	Quality assessment
Buntrock et al. [41, 42]	*N* = 406; German general pop. (no MDD on SCID). Aged 18+ years (73.9% female)	i. iCBT ii. Psychoed.	Automated SMS reminders, 2 h online trainer feedback	6 × 30 min sessions (3–6 weeks). Behavioural and problem-solving therapy.	6 weeks (90.1%) 6 months (80.0%) 12 months (82.3%) Mean sessions: 5.84	Symptom reduction (CES-D). MDD incidence.	6-week BG ES: Cohen’s *d* = 0.69 (F_1,403_ = 54.104, *p* < .001). 6-month BG ES: Cohen’s *d* = 0.28 (F_1,402_ = 9.240, *p* = .003). There was a significant difference in MDD incidence rates over 12 months favouring the intervention group (hazard ratio = 0.59, 95% CI: 0.42–0.82; *p* = .002).	23
Christensen et al. [43]	*N* = 558; Australian Internet users (No anxiety disorder on MINI). Aged 18–30 years (80.6% female)	i. iCBT + Psychoed. (A) ii. A + telephone reminders iii. A + email reminders iv. Placebo website (B) v. B + telephone reminders	Differing reminder conditions, 2-min/week. No therapeutic content.	10 × 10 weekly sessions. Mindfulness-focussed CBT for anxiety (e-couch).	10 weeks (64.5%) 6 months (54.3%) 12 months (47.3%) Mean sessions: (i) 3.7; (ii) 7.3; (iii) 5.5; (iv) 3.7; (v) 8.3.	Symptom reduction (GAD-7). GAD incidence.	Significant time effects for each of the three follow-ups. No significant group × time effects for any comparison. Overall, indicated prevention of GAD was deemed not effective	21
Clarke et al. [46]	Relevant subgroup: *n* = 63; US HMO member adults (CES-D < 20). Aged 18+ years (75.6% female)	i. iCBT + Psychoed. ii. Usual care	No support.	7 chapters. CBT skills program (focusing on the cognitive restructuring techniques).	4 weeks (52.8%) 8 weeks (65.2%) 16 weeks (65.5%) 32 weeks (59.2%) Mean logins: 2.6	Symptom reduction (CES-D).	Significant reduction in symptoms in intervention participants compared to control at the 16-week (BG ES: Cohen’s *d* = 0.17, *p* < .05) and 32-week (BG ES: Cohen’s *d* = 0.48, *p* < .01) follow-up.	16
Cukrowicz et al. [47]	*N* = 152; US undergrad. (BAI ≤ 18; BDI ≤ 19). 95% aged 18–21 years (73.7% female)	i. Psychoed. + CBT ii. Psychoed.	Facilitated session.	6 × 20 min segments (1 laboratory session). Situational analysis-focussed CBT.	2 months (90.3%)	Symptom reduction (BAI & BDI).	BAI, BG ES: Cohen’s *d* = 0.24 (F_1,145_ = 7.84, *p* < .01). BDI, BG ES: Cohen’s *d* = 0.27 (F_1,145_ = 9.64, *p* < .01. Additional significant reductions on PANAS and STAI-S, and Reliable Change Index.	17
Imamura [44, 52]	N = 762; Japanese workers (No past month MDD on WHO-CIDI). Aged 18+ years (16.1% female)	i. iCBT ii. Stress reduction tips email	Email reminders. Homework feedback from clinical psychologist.	6 × 30 min sessions (6 weeks). CBT skills program (self-monitoring, cognitive restructuring, assertiveness, problem solving, and relaxation).	3 months (79.5%) 6 months (77.7%) 12 months (67.1%) Mean sessions: 4.53	Symptom reduction (BDI-II). MDE incidence.	3-month BG ES: Cohen’s *d* = −0.14, 95% CI: −0.30 to 0.02 (t _621.35_ = − 1.99, *p* < .05). 6-month BG ES: Cohen’s *d* = −0.16, 95% CI: −0.32 to 0.00 (t _621.35_ = − 1.99, *p* < .05). 12-month BG ES: Cohen’s *d* = −0.08, 95% CI: −0.26 to 0.09 (t _610.33_ = − 1.42, *p* = .16). Significant reduction in MDE incidence in intervention compared to control at 12-month (Log-rank *χ* ^*2*^ = 7.04, *p* < .01) but not 6-month (Log-rank *χ* ^*2*^ = 3.26, *p* = .07).	24
Levin et al. [48]	Relevant subgroup: n = 43; US undergrad. (DASS in normal range). Aged 18–21 years (53.9% female)	i. ACT ii. Waitlist	Email reminders.	2 sessions. Youth-focussed ACT program.	3 weeks (79.5%) 6 weeks (77.7%)^a^ 92% completed program	Symptom reduction (DASS).	No significant between group differences were observed on depression, anxiety of stress among the non-distressed subgroup (*p* > .10).	19
Lintvedt et al. [49]	Relevant subgroup: n = 52; Norwegian undergrad. (subclinical: CES-D). Aged 18+ years (53.9% female)	i. Psychoed. + iCBT ii. Waitlist	Weekly automated assignments.	5 weekly modules. CBT, interpersonal therapy, relaxation self-help program (Moodgym) & psychoed. Program (Bluepages)	8 weeks (68.0%) Mean modules: 2–3	Symptom reduction (CES-D).	There was a significant increase in depressive symptoms for the subclinical control group compared to the intervention group (*F*_1,24_ = 6.86, *p* < .05; Hedges *g* = 0.61).	22
Morgan et al. [50]	*N* = 1326; English speaking gen. Pop. (subclinical: PHQ-9) Aged 18–79 years (77.6% female)	i. Self-help emails ii. Psychoed. emails	No support.	2 emails/week (6 weeks). Persuasive framing, tailoring, goal setting, limiting cognitive load.	3 weeks (54.8%) 6 weeks (42.9%) 95.6% received the emails	Symptom reduction (PHQ-9).	There was a small significant difference in depression symptoms in intervention group compared to control (*d* = 0.17, 95% CI: 0.01 to 0.34). There was a lower, although non-significant, risk of major depression in the active group.	20
Musiat et al. [51]	Relevant subgroup: n = 859; UK tertiary students (low risk on the SURPS). Aged 18–57 years (70.5% female)	i. iCBT ii. Student life program	No support.	5 × 30 min modules Personality trait-driven CBT program (PLUS)	6 weeks (49.7%)^a^ 12 weeks (38.3%) 47% completed a module	Symptom reduction (PHQ-9; GAD-7).	Significant intervention effects were found in the high risk group but for those at low risk no significant change was detected in PHQ-9 (*p* > .999) or GAD-7 (*p* = .415)	21
Spek et al. [40, 45]	*N* = 301; Dutch older general pop. (no MDD on WHO-CIDI). Aged 50–75 years (63.5% female)	i. iCBT ii. group CBT iii waitlist	No support.	8 sessions (8 weeks) Psychoed. and CBT (Coping with depression)	10 weeks (60.1%) 12 months (63.1%) Mean modules: 5.5	Symptom reduction (BDI-II).	Significant intervention effects were found in both intervention groups compared to control. No differences were found between interventions (Internet vs control: post-treatment BG ES: Cohen’s *d* = 0.55; 12-month BG ES: Cohen’s *d* = 0.53).	24

*MDD* major depressive disorder, *MDE* major depressive episode, *Psychoed.* psychoeducation, *iCBT* Internet Cognitive Behavioural Therapy, *SCID* Structured Clinical Interview for DSM, *TAU* treatment as usual, *MINI* Mini-International Neuropsychiatric Interview, *HMO* Health Maintenance Organization, *BDI-II* Beck Depression Inventory, *BAI* Beck Anxiety Inventory, *WHO-CIDI* World Health Organisation Composite International Diagnostic Interview, *PHQ-9* Patient Health Questionnaire, *GAD-7* Generalized Anxiety Disorder–7-item scale, *BG ES* between-group effect size, *ACT* Acceptance and Commitment Therapy, *CES-D* Center for Epidemiological Studies-Depression, *DASS* Depression Anxiety Stress Scale; *undergrad.* Undergraduate, *PANAS* Positive and Negative Affect Schedule, *STAI-S* State-Trait Anxiety Inventory

^a^Results not reported at this follow-up point

Four of the 10 independent samples utilized a clinical diagnostic tool to rule out current mental health diagnosis at baseline [42–45]. Five of the remaining six used recommended subclinical cut-offs on validated self-reported measures of depression and anxiety [46–50]. The final study used a cut-off on a self-report measure of mental disorder risk to determine the subclinical subgroup relevant to this review [51].

Tertiary students were the sample population of four studies from the US [47, 48], the UK [51], and Norway [49]. The remaining studies targeted general population adults in the US [46], Australia [43, 50], and Germany [42], Japanese workers [44] and older Dutch citizens [45].

### Types of intervention

Symptom reduction was the primary outcome in all studies, with true prevention in the form of reduced 12-month *incidence* rates an outcome measure in only two studies [41, 52]. Depression was the sole focus in six studies [42, 44–46, 49, 50], anxiety in one study [43] and both conditions were primary outcomes in the remaining three studies [47, 48, 51]. Despite self-selection biases inherent to (opt-in) research trials, five trials could be considered universal prevention [43, 44, 46–48], in that they recruited from general, healthy populations and had no low-end cut-off for inclusion. Four trials were indicated prevention [42, 45, 49, 50]—as these recruited symptomatic groups—and one trial was selective prevention [51], targeting those with specific personality traits.

With the exception of Acceptance and Commitment Therapy (ACT) [48] and unspecified self-help emails [50], the interventions were all labelled as Cognitive Behavioural Therapy (CBT). Morgan and colleagues [50] claimed the self-help emails were aimed at persuasive framing, tailoring, goal setting, and limiting cognitive load. Buntrock and colleagues [42] used a behavioural and problem-solving form of CBT. Personality traits were the focus of Musiat and colleagues’ [51] CBT program, while self-monitoring, cognitive restructuring, assertiveness, problem solving, and relaxation were listed as the major component of the remaining programs. Four studies were completely unsupported [45, 46, 50, 51], two included automated email support only [48, 49]. The remaining studies utilised a variety of non-clinical reminders (e.g. email, telephone [43]), SMS/email reminders in combination with optional homework feedback [42, 44] and a facilitated session [47].

### Quality

The studies ranged from “fair” to “good” in quality (16–24) (Table 2). Thus, all were included in the meta-analysis. In three [46, 48, 49] of the five studies where only a subgroup met review criteria, sample size was small. Half the studies [40, 42–44, 46] had medium to long-term follow-ups (at least 6 months) and the majority of studies utilised some form of active-control condition [42–45, 47, 50, 51].

### Effects at post-intervention outcomes

**Fig. 2:**
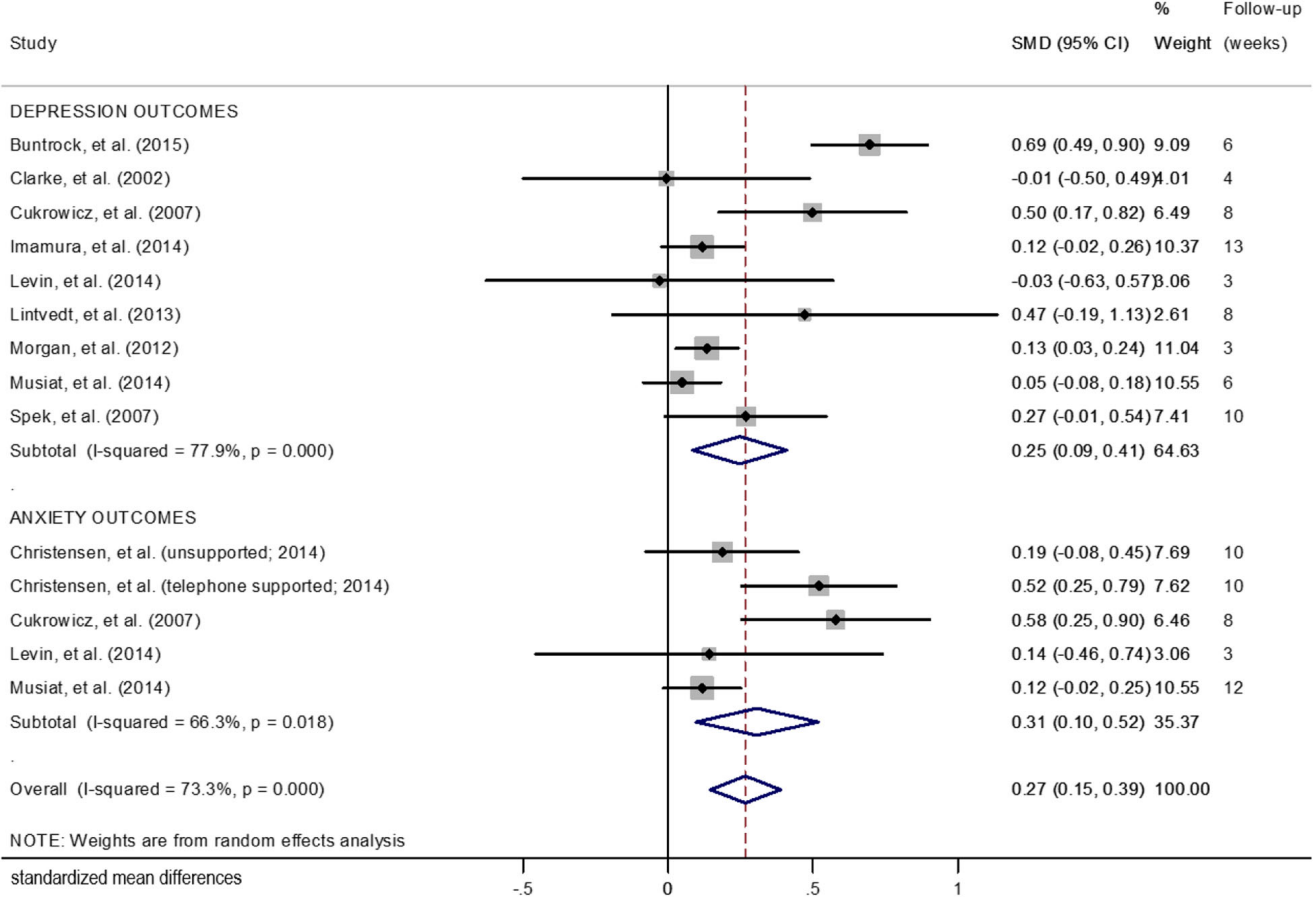
Effects of eHealth prevention interventions on symptoms (post-intervention)

The SMDs for symptom reduction immediately after the interventions occurred is presented in Fig. 2. For depression outcome studies, the overall mean difference between the intervention and control groups was 0.25 (95% CI: 0.09, 0.41; *p* = 0.003). A high degree of heterogeneity was detected (Q = 36.35; I^2^ = 77.9%; *p* ≤ 0.001). For anxiety outcome studies, the overall mean difference between the intervention and control groups was 0.31 (95% CI: 0.10, 0.52; *p* = 0.004). A moderate to high degree of heterogeneity was detected (Q = 11.86; I^2^ = 66.3%; *p* = 0.018).

### Effects at follow-up (at least 6-month)

**Fig. 3:**
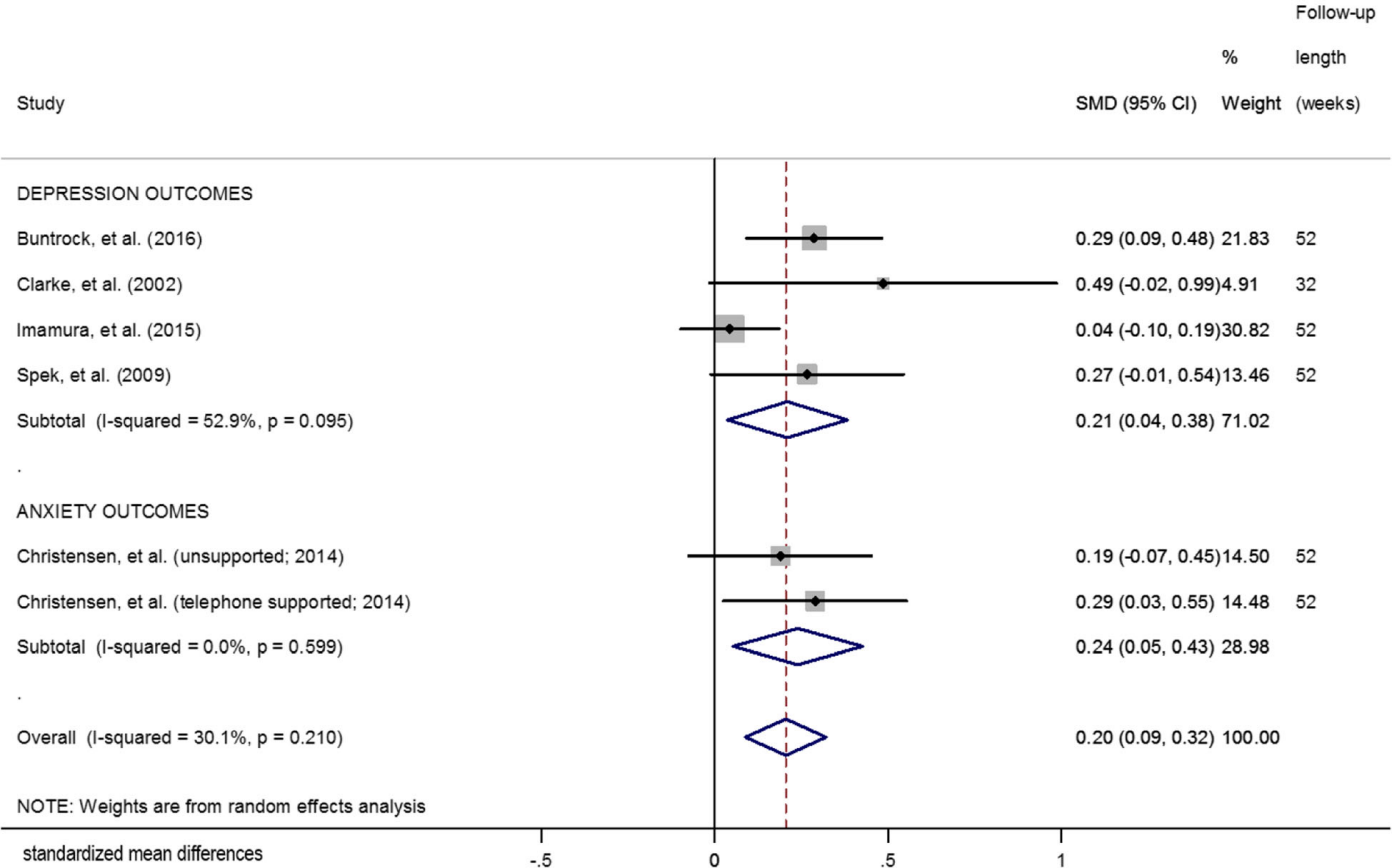
Effects of eHealth prevention interventions on symptoms (at least 6-month follow-up)

The SMDs at follow-up and the pooled mean effect size for the four depression studies that included a follow-up of at least 6-months are presented in Fig. 3. The overall mean difference between intervention and the control groups was 0.21 (95% CI: 0.04, 0.38; *p* = 0.02), indicating a positive effect. A moderate, though not statistically significant, degree of heterogeneity was present in this analysis (Q = 6.37; I^2^ = 52.9%; *p* = 0.11). Three depression studies reported data at 12-month follow-up [40, 41, 52]. The overall pooled RR was 0.42 (95% CI: 0.13, 1.35, *p* = 0.15). A high degree of heterogeneity was detected (Q = 3.60; I^2^ = 72.2%; *p* = 0.06).

Only one study with anxiety outcomes included a follow-up longer than 6 months [43]. The overall mean difference between the intervention and control groups was 0.24 (95% CI: 0.05, 0.43, *p* = 0.01). At 6-months the overall RR for this study (iCBT arms compared to control arms) was 1.42 (95% CI: 0.537, 3.727; *p* = 0.482).

### Secondary analyses

**Fig. 4:**
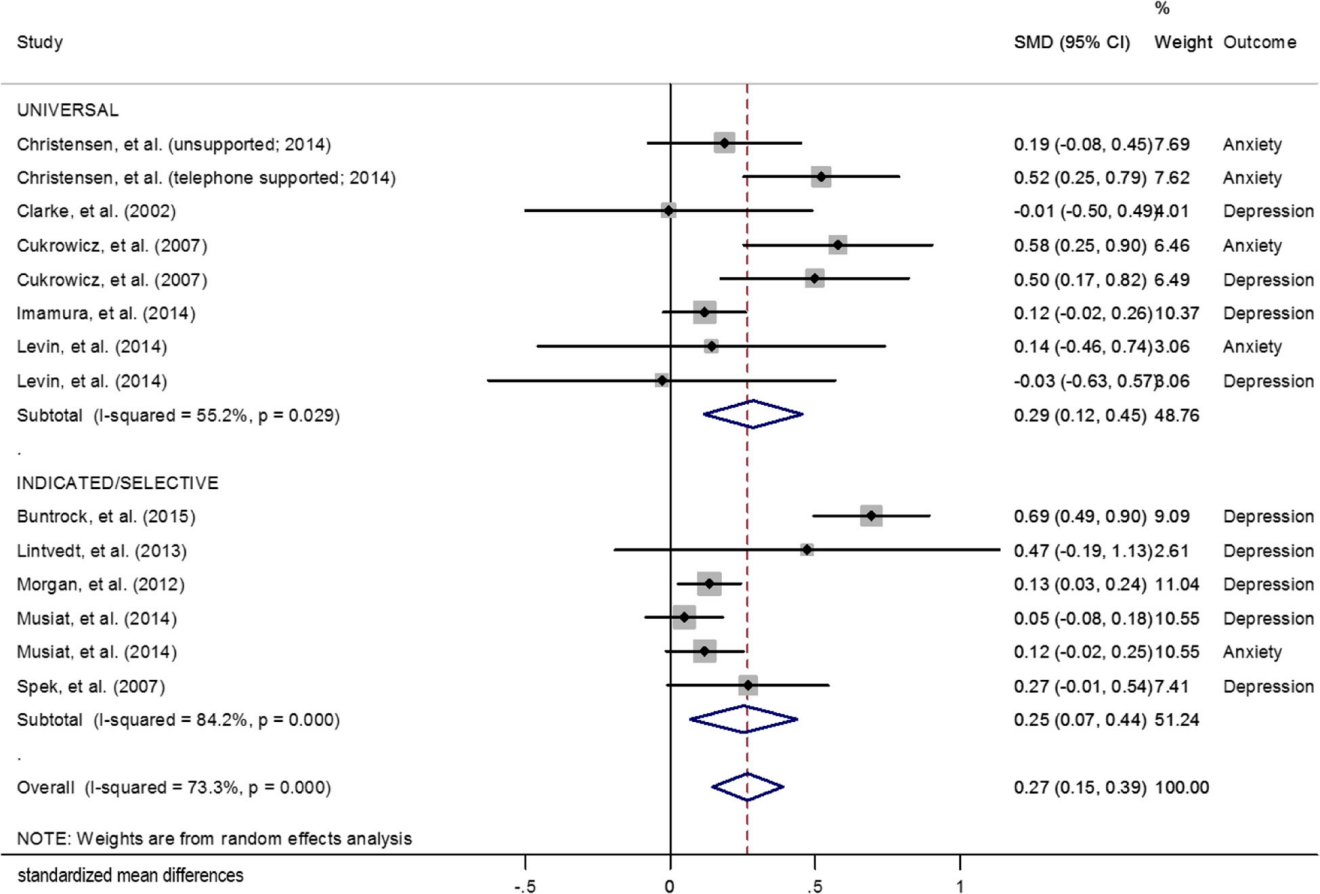
Effects of different types of eHealth prevention interventions on symptoms

The post-intervention SMDs and the pooled mean effect size for both universal and indicated/selected prevention interventions are presented in Fig. 4. The pooled mean effect size for the studies of universal prevention programs was 0.29 (95% CI: 0.12, 0.46, *p* = 0.001). A moderate degree of heterogeneity was detected (Q = 15.62; I^2^ = 55.2%; *p* = 0.03). The pooled mean effect size for the indicated/selective prevention programs was almost identical at 0.25 (95% CI: 0.07, 0.44, *p* = 0.007). A high degree of heterogeneity was detected (Q = 31.7; I^2^ = 84.2%; *p* ≤ 0.001).

### Analysis of publication bias

There was no evidence asymmetry upon inspection of funnel plots. However, due to the limited number of studies included in the analysis, Egger’s linear regression model was also used. The Egger’s regression test for asymmetry suggested that there was no significant publication bias for depression outcome studies (*p* = 0.429) anxiety outcome studies (*p* = 0.325), universal prevention (*p* = 0.622), or selective/indicated prevention (*p* = 0.331).

## Discussion

This is the first systematic review and meta-analysis examining randomized controlled trials of eHealth interventions aimed at preventing depression and anxiety in the general population. Our results were drawn from ten randomised controlled studies with fair to good quality. Results indicate that a range of different cognitive behavioural programs produce small, but overall positive effects on symptom reduction for anxiety and depression, at both an indicated/selective and universal prevention level. Although the effect sizes among eHealth prevention interventions appear to be smaller than that reported for face-to-face prevention interventions [53], eHealth has potential for more reach with fewer resources. Indeed, eHealth technologies may be able to overcome a range of financial, geographic, and time barriers that have previously existed in this area. As such, these symptom effects may have considerable impact at a population level. Furthermore, considering the prevalence [54] and impairment associated with even subsyndomal disorders [55–57], this effect has the potential to make meaningful change. Prevention interventions are never likely to produce large individual effect sizes, as they are delivered to the mass populations who are not unwell, but when translated to a population level, the overall impact of these small effects can be substantial and result in dramatic improvements in public health outcomes [58]. The eHealth prevention interventions included in this meta-analysis produced similar pooled mean effect sizes for both depression and anxiety outcomes. This may be a consequence of the overlap in symptoms between these conditions [59] and the lack of diagnostic outcomes used. The majority of studies utilised some form of CBT, with behavioural, mindfulness, and problem-solving components often a major focus. It is consistently shown that eHealth interventions that include some form of guidance are associated with larger effect sizes than those that do not [60], interestingly, the degree of support provided for this sample was relatively minimal, and the heterogeneous nature of this support prohibited further exploration. Although reminders were consistently used in the studies reviewed, few provided any form of therapist feedback. This is of particular relevance when considering adherence rates and overall effectiveness, as subclinical populations (such as those studies here) have been said to lack intrinsic motivation for completing the programs evoked by a diagnostic treatment imperative [61]. Despite the relatively minimal guidance provided among the prevention studies, program adherence was moderate across the studies and not dissimilar to that of clinical studies [62, 63].

Overall, similar effectiveness was found across the universal and selected/indicated eHealth prevention interventions. Although there is some evidence to suggest selected/indicated interventions might be more successful that universal approaches [64, 65], the majority of published studies in this area utilise school-based populations. Furthermore, many universal interventions may not be appropriately powered to find such effects as the sample size required for universal prevention can be overwhelming [66, 67]. The findings presented here are in line with those of Jane-Llopis and colleagues [68], and cast some doubt on the categorical superiority of selected/indicated interventions within the general population.

What is evident from this review is not only the lack of true eHealth prevention trials published in the literature [17, 69], but of those trials that do specifically target a non-clinical sample (be it universal, selective, or indicated prevention), the primary (and in most cases the only) relevant outcome is short-term symptom reduction rather than a decrease in disorder incidence. Three depression studies [40, 41, 52] and one anxiety study [43] reported medium to long-term follow-up, with 12-month incidence rates reported for only two studies [41, 52]. While each of the studies that examined depression diagnosis as an outcome reported significant differences at 12-months, when both were included in a meta-analysis the overall effect fell short of statistical significance. As such, there is inadequate evidence to suggest that eHealth interventions have an effect on long-term outcomes (especially for anxiety) and disorder incidence rates.

One of the key issues in conducting prevention trials is the sample sizes required to gain sufficient statistical power. For instance, it has been suggested that to demonstrate that a universal prevention program could reduce the rates of new onset depression over 12 months by 15%, the number of subjects required amounts to tens of thousands [13]. Furthermore, accurate diagnostic-based prevention within automated eHealth interventions is rare as this requires assessment contact with research staff, diminishing some key benefits of eHealth interventions (anonymity, low cost). Finally, the mental illness prevention field is relatively new [15], and although there has been promising results on the feasibility of prevention as a way of reducing the incidence and overall burden of common mental disorders at a community level, eHealth is also in its infancy [17].

The main strengths of this review include a detailed systematic search strategy and the objective assessment of the methodological rigor of each included study. Despite these strengths, there are a number of other limitations to this review. First, due to the limited number of studies, comparison of different theoretical approaches, different forms of prevention (universal, selective, or indicated), and other elements was difficult. Indeed, although this meta-analysis reported similar effectiveness among the universal and indicated/selective eHealth prevention interventions, there are classification issues that make interpretation difficult. For instance, due to the opt-in nature of research trials it is possible that the universal programs do not accurately reflect true universal prevention, as those taking part in such studies may have had symptoms of concern, which provided motivation for initial engagement. Conversely, some selective interventions of non-generalised populations were excluded from the review (e.g., postnatal, chronic pain), as they are often associated with fundamental differences which are likely to preclude the translation of such programs to other populations. Additionally, it would have been advantageous to make specific comparisons of differing theoretical approaches in order to determine which held most effectiveness in prevention, again limited numbers precluded this exploration. Similarly, the limited number of interventions and different types of techniques used may explain the heterogeneity observed in the data. The high levels of heterogeneity suggest treatment effect estimates vary among the studies, which may be due to different intervention content, engagement or difference in the populations sampled. While we utilised random effect models to help account for this, the remaining heterogeneity places some limitations on the interpretation of the pooled effect size, which may not be an accurate summary of the effects in all interventions. Secondly, as mentioned, self-report measures were the primary outcome in all studies, and therefore, conclusions are largely limited to reductions in symptoms rather than clinical diagnosis. Thirdly, as study populations were randomized, we conducted the meta-analysis under the assumption that there was no pre-test difference in scores for the control and treatment groups. Most studies reviewed tested for this and reported that no significant differences were present in the pre-test scores; however, in studies that did not, or where subgroups were used, this assumption may not hold [47]. Finally, as non-English publications and non-published articles were excluded, there is a possibility that such studies published in other languages or in the grey literature may have not been identified.

Although beyond the scope of this review it is important to note that one final limitation in evaluating effective prevention in this area is that of the search strategy [70]. The search specifically aimed to look at mental health disorders, however, this may have omitted relevant studies of wellbeing, or other less diagnostic studies (e.g., [71]). In short, these studies tend to measure reductions in the risk of illness by measuring symptoms of illness. In doing so the prevention techniques used were all ‘therapeutic’ (i.e., treatments intended to relieve or heal a disorder). This is a limitation across the clinical field of mental health prevention, as conditions lie on a spectrum and there is no accepted modifiable risk marker that is indicative of future illness. It would be useful to better understand the impact of CMD prevention interventions that do not target symptoms and whether other (non-therapeutic) techniques are effective (e.g., [72]). However, there is scepticism around the efficacy of wellbeing interventions as prevention [73].

Again, although beyond the scope of this review, a popular setting for prevention interventions is within school settings [74–77], due to the advantages of access and ease with which such programs can be incorporated into the curriculum. Modern workplaces are becoming increasingly aware of the cost and impact of mental illness at an employee level, and initiatives in this area may lead to the workplace becoming an equivalent location where working-age adults can be engaged [33, 78].

## Conclusions

In conclusion, this systematic review and meta-analysis found small but positive effects of eHealth prevention interventions for anxiety and depression. However, there is inadequate evidence on the medium to long-term effect of such interventions, and importantly, on the reduction of incidence of CMD. As little variation existed in the theories and techniques used it would be useful to explore other preventive strategies in eHealth delivery, (e.g. behavioural or mindfulness approaches), particularly those that have shown good effects in face-to-face sessions. Further work is needed to ascertain appropriate settings for such prevention work and to further explore the impact of eHealth psychological interventions on long-term incidence rates.

## Abbreviations

95% CI: 95% confidence internal
ACT: Acceptance and Commitment Therapy
BAI: Beck Anxiety Inventory
BDI-II: Beck Depression Inventory
BG ES: Between-group effect size
CBT: Cognitive Behavioural Therapy
CES-D: Center for Epidemiological Studies-Depression
CMD: Common mental disorders
DASS: Depression Anxiety Stress Scale
GAD-7: Generalized Anxiety Disorder–7-item scale
HMO: Health Maintenance Organization
iCBT: Internet Cognitive Behavioural Therapy
MDD: Major depressive disorder
MDE: Major depressive episode
MINI: Mini-International Neuropsychiatric Interview
PANAS: Positive and Negative Affect Schedule
PHQ-9: Patient Health Questionnaire-9
Psychoed.: Psychoeducation
REM: Random effects model
RR: Risk ratios
SCID: Structured Clinical Interview for DSM
SE: Standard error
SMD: Standardized mean differences
SMS: Short messaging service
STAI-S: State-Trait Anxiety Inventory
TAU: Treatment as usual
UK: United Kingdom
undergrad.: Undergraduate
US: United States
WHO-CIDI: World Health Organisation Composite International Diagnostic Interview
